# Telephone-based Frontal Assessment Battery (t-FAB): standardization for the Italian population and clinical usability in neurological diseases

**DOI:** 10.1007/s40520-022-02155-3

**Published:** 2022-06-14

**Authors:** Edoardo Nicolò Aiello, Veronica Pucci, Lorenzo Diana, Aida Niang, Alice Naomi Preti, Adriana Delli Ponti, Gaia Sangalli, Stefano Scarano, Luigi Tesio, Stefano Zago, Teresa Difonzo, Ildebrando Appollonio, Sara Mondini, Nadia Bolognini

**Affiliations:** 1 PhD Program in Neuroscience, School of Medicine and Surgery, University of Milano-Bicocca, Via Cadore 48, 20900 Monza, Italy; 2 Dipartimento di Filosofia, Sociologia, Pedagogia e Psicologia Applicata (FISPPA), University of Padova, Padua, Italy; 3 Human Inspired Technology Research Centre (HIT), University of Padova, Padua, Italy; 4 Department of Psychology, University of Milano-Bicocca, Milan, Italy; 5 Neurology Unit, Ospedale Maggiore Policlinico, Fondazione IRCCS Cà Granda, Milan, Italy; 6 Neuropsychological Laboratory, IRCCS Istituto Auxologico Italiano, Milan, Italy; 7 Department of Neurorehabilitation Sciences, IRCCS Istituto Auxologico Italiano, Milan, Italy; 8 Department of Biomedical Sciences for Health, University of Milan, Milan, Italy; 9 Neurology Section, School of Medicine and Surgery, University of Milano-Bicocca, Monza, Italy

**Keywords:** Telephone-based, Cognitive screening, Frontal assessment battery, Teleneurology, Executive functioning, Stroke, Neurological disease

## Abstract

**Background:**

Despite the relevance of telephone-based cognitive screening tests in clinical practice and research, no specific test assessing executive functioning is available. The present study aimed at standardizing and providing evidence of clinical usability for the Italian telephone-based Frontal Assessment Battery (t-FAB).

**Methods:**

The t-FAB (ranging 0–12), comprising two subtests, has two versions: one requiring motor responses (t-FAB-M) and the other verbal responses (t-FAB-V). Three hundred and forty-six Italian healthy adults (HPs; 143 males; age range = 18–96 years; education range = 4–23 years) and 40 participants with neurological diseases were recruited. To HPs, the t-FAB was administered along with a set of telephone-based tests: MMSE, verbal fluency (VF), backward digit span (BDS). The in-person version of the FAB was administered to both HPs and clinical groups. Factorial structure, construct validity, inter-rater and test–retest reliability, t-FAB-M vs*.* t-FAB-V equivalence and diagnostic accuracy were assessed. Norms were derived via Equivalent Scores.

**Results:**

In HPs, t-FAB measures yielded high inter-rater/test–retest reliability (ICC = .78–.94), were internally related (*p* ≤ .005) and underpinned by a single component, converging with the telephone-based MMSE, VF, BDS (*p* ≤ .0013). The two t-FAB versions were statistically equivalent in clinical groups (*p*s of both equivalence bounds < .001). Education predicted all t-FAB scores (*p* < .001), whereas age only the t-FAB-M score (*p* ≤ .004). t-FAB scores converge with the in-person FAB in HPs and clinical groups (*r*_*s*_ = .43–.78). Both t-FAB versions were accurate in discriminating HPs from the clinical cohort (AUC = .73-.76).

**Discussion:**

The t-FAB is a normed, valid, reliable and clinically usable telephone-based cognitive screening test to adopt in both clinical and research practice.

**Supplementary Information:**

The online version contains supplementary material available at 10.1007/s40520-022-02155-3.

## Introduction

Telephone-based cognitive screening (TBCS) is relevant to clinical and experimental telemedicine, as allowing first-level neuropsychological evaluations when in-person access to clinics is not possible due to logistical [1, 2], geographical [3], economical [4] and safety reasons [5]. TBCS also facilitates the implementation of epidemiological/population-based studies [6], decentralized clinical trials [7] and prevention campaigns [8]. Moreover, at variance with other remote assessment media (e.g., videoconference), the telephone proves to be well accepted by receivers of different socio-demographic backgrounds as requiring minimal digital and health literacy [9, 10], being also judged by users as adequate to meet clinical/research questions [9]. It has to be then noted that, as other telehealth practices, TBCS has the potential to reduce the psychosocial burden related to in-person healthcare, weighting not only on patients, but also on their caregivers and professionals carers, which has significantly increased since the onset of the COVID-19 pandemic [11, 12].

Although several TBCS tools for the assessment of global cognitive status have been developed in recent years [6], no standardized test selectively screening executive functions is available, especially in Italy [13, 14]. Indeed, fully standardized Italian TBCS tests are currently limited to multi-domain screening tests for global cognition [2, 15].

Nevertheless, executive deficits feature a variety of neurological diseases affecting cortical and subcortical frontal circuitries [16] and convey relevant information towards differential diagnosis and prognosis [17]. Moreover, sub-clinical or mild dysexecutive features are highly prevalent in healthy elderlies due to the anatomo-functional changes of frontal networks resulting from physiological aging [18, 19], which were shown to negatively impact on daily living functional outcomes [17]. Screening for executive dysfunctions is thus crucial in both neurology and geriatrics, and the availability of standardized TBCS tests for frontal-executive dysfunctions should be part of telemedicine [14], especially when dealing with elderly clinical populations. Indeed, aging individuals often come with a range of frailties that would prevent or delay their access to outpatient in-person clinics, due to both logistical (e.g., motor disabilities) and health safety reasons (e.g., an increase exposure to SARS-CoV-2 infection) [3, 8, 14]. Such instruments would be also useful to be adopted as outcome measures/endpoints within large-scale studies or decentralized clinical trials in neurological and geriatric populations with frontal-executive impairments [20].

Among frontal-executive screeners, the in-person version of the Frontal Assessment Battery (FAB) [21] represent one of the most psychometrically and diagnostically sound tests worldwide [22]. In Italy, the FAB has been shown to come with optimal psychometrics, diagnostics and clinical usability evidence [18, 19, 23, 24].

Therefore, this study aimed at standardizing, in the Italian population, a telephone-based version of the FAB (t-FAB), also exploring its clinical usability in different neurological diseases.

## Methods

### Participants

The normative sample comprised 346 Italian healthy participants (HPs) from different regions of Italy. Inclusion criteria were: (1) negative anamnesis for neurological/psychiatric disorders, not being (2) under active psychotropic medications or (3) in uncompensated/severe metabolic/internal conditions, (4) not having system/organ failures, (5) presenting with uncorrected hearing deficits. Medical history for all participants was collected through a semi-structured interview. HPs were recruited via the authors’ personal acquaintances and advertising at the University of Milano-Bicocca and the University of Padova.

A total of 40 participants affected by neurological diseases were consecutively recruited at two neuropsychology services in Northern Italy. Inclusion criteria for clinical groups were a neurologist-posed clinical diagnosis of a given target disease based on current diagnostic criteria and supported by neurological, neuroradiological and neuropsychological examinations. Ten participants with ischemic/hemorrhagic stroke presenting with unilateral, cortical/subcortical hemispheric lesion (5 right and 5 left brain-damaged) were recruited at the neuropsychology service of IRCCS Istituto Auxologico Italiano, Milano. Five participants affected with hypokinetic extra-pyramidal disorders (three with Parkinson’s disease [25], and three with atypical parkinsonisms [26]), 20 with small vessel disease (SVD) [27] and 4 with a mixed, atrophic-vascular dementia (MD) [28] were recruited at the neuropsychology service of Fondazione IRCCS Ca’ Granda Ospedale Maggiore Policlinico, Milano. The full selection process of participants with neurological conditions is shown as follows (“Results”, paragraph “Standardization”).

The study was approved by the Ethical Committees of the University of Milano-Bicocca, Milano (ID: RM-2021–382, 19/02/2021), the University of Padova, Padova (ID: 4107, 19/02/2021) and IRCCS Istituto Auxologico Italiano, Milano (ID: 25C122, 18/05/2021). Participants provided their informed consent. Data collection for HPs started in March 2021 and ended in January 2022, whereas that for clinical populations started in July 2021 and ended in January 2022.

### Materials

The t-FAB was adapted from Aiello et al.’s [24] version of the FAB. The in-person FAB total score ranges from 0 to 18 and it comprises 3 subtests (each ranging from 0 to 6), each including two tasks: *Conceptualization*, *Mental flexibility* (language-mediated executive functions, FAB-1), *Motor programming* and *Sensitivity to interference* (motor-mediated executive functions, FAB-2) and *Inhibitory control* and *Environmental autonomy* (inhibition; FAB-3) [24]. In the t-FAB, *Conceptualization* and *Mental flexibility* tasks were the same of the in-person version, whereas *Programming* and *Environmental autonomy* tasks were removed. *Sensitivity to interference* and *Inhibitory control* tasks were modified to adapt to the telephonic administration (i.e., for participants’ responses to be audible by the examiner through the telephone). Two versions of both *Sensitivity to interference* and *Inhibitory control* tasks, different as to their response modality, were developed: the first requiring the examinee to provide a motor response (t-FAB-M; e.g., “*tap once on the table when I tap twice*”), the second to provide a verbal one (t-FAB-V; e.g., “*say «one» when I say «two»*”). In case of lateralized sensorimotor deficits, participants with neurological diseases were asked to use their unaffected hand. Both t-FAB-M and t-FAB-V versions of *Sensitivity to interference* and *Inhibitory control* tasks were administered to all participants (order: FAB-M first). In addition, each t-FAB is divided into two subtests: t-FAB-1 (*Conceptualization* plus *Mental flexibility*) and t-FAB-2-M/-V (*Sensitivity to interference* plus *Inhibitory control*). The total t-FAB score of both versions ranged 0–12.

The full t-FAB protocol is provided in Supplementary Material 1. All the above adaptations were jointly conceptualized and operationalized by an author board that comprised a neurologist (IA), three neuropsychologists (SZ, SM, NB), three neuroscientists (ENA, VP, LD) and two physiatrists expert in psychometrics (LT, SS), all with extensive expertise in the field of cognitive test standardization. No disagreement emerged in this first phase, and the protocol was subsequently approved without reservation by the remaining authors [30, 31].

For assessing construct validity, the following tests were remotely administered to the following HP subsamples:

- phonemic, semantic and alternate verbal fluency tests (PVF; SVF; AVF) (HP subsample of *N* = 330: 138 males, 192 females; age: 48.39 ± 18.81 years, 18–96; education: 13.46 ± 3.75 years, 4–23), along with the derived Cognitive Shifting Index (CSI), a measure of cognitive flexibility [29];
- the Italian telephone-based Mini-Mental State Examination (Itel-MMSE; HP subsample of *N* = 253: 99 males, 154 females; age: 47.11 ± 18.67 years, 18–96; education: 13.7 ± 3.65 years, 4–22) [30; 31];
- a backward digit span (BDS) task [32] (HP subsample of *N* = 270: 110 males, 160 females; age: 47.24 ± 18.33 years, 18–96; education: 13.67 ± 3.63 years, 4–22), comprising two scores: the longer recalled digit sequence (a measure of working memory capacity; BDS-WM) and the total number of recalled sequences (a measure of sustained attention during task execution; BDS-T) [33].

To assess invariance between the t-FAB and its in-person version, 50 HPs (19 males, 31 females; age: 46.22±16.83 years, 20-86; education: 13.82±3.53 years, 5-23) underwent the paper-and-pencil FAB 48 hours before (*N*=25) or after (*N*=25) the remote assessment to rule out carry-over effects. A different subgroup of 20 HPs (11 males, 9 females; age: 42.6±17.9 years, 19-80; education: 12.1±3.46 years, 5-16) were administered the FAB either 14 days before (*N*=10) or after (*N*=10) the t-FAB.

For test–retest reliability, the t-FAB was administered two times, at a 30-day distance, to 27 HPs (12 males, 15 females; age: 47.56 ± 21.14 years, 23–85; education: 14.74 ± 4.42 years, 5–21), whereas, for inter-rater reliability, 25 protocols (8 males, 17 females; age: 45.2 ± 17.67 years, 21–74; education: 14.4 ± 3.19 years, 8–20) were simultaneously scored by two independent raters blinded to each other’s decisions.

All the above HP subsamples were randomly selected through random number tables from the whole normative sample.

Participants with neurological diseases also underwent the in-person FAB, delivered 7–14 days before t-FAB administration.

Before telephone-based testing, all participants underwent a detailed sound-check to ensure a good quality of the call [15].

All tests were administered to both HPs and individuals with neurological diseases by licensed psychologists with thorough expertise in neuropsychological assessment and/or trainees neuropsychologists (ANP, ADL, GS, and TD). Testers underwent a thorough administration training performed by the corresponding author (ENA). Statistical analyses were performed by four neuroscientists with thorough expertise in cognitive data analyses (ENA, VP, LD, and ANP) and approved by the whole author panel.

### Statistical analyses

Analyses were run via SPSS 27 (IBM Corp., 2020), R 4.1.0 (https://cran.r-project.org/) and jamovi 1.6.23 (https://www.jamovi.org/). The 2-tailed significance level was set at α = 0.05 and Bonferroni correction applied to multiple comparisons whenever necessary.

Either Pearson’s or Spearman’s technique was adopted to test for construct validity in HPs based on data distributing normally or not, respectively (i.e., skewness and kurtosis values <|1| and |3|, respectively) [34]. Test–retest and inter-rater reliability in HPs were assessed through intra-class correlations. Factorial structure in HPs was explored through a principal component analysis (PCA) by entering each task separately for the t-FAB-M and t-FAB-V.

According to the recommendations by Hobart et al*.* [35], which relate to health measurement studies for clinical neurology, the minimum sample size for all the aforementioned reliability and validity analyses in HPs were set at *N* = 20 and *N* = 80, respectively, except for the PCA, for which a *N* = 100 was deemed as sufficient according to guidelines delivered by Kyriazos [36].

Equivalence between the t-FAB-M and t-FAB-V was assessed in HPs through a two one-sided test procedure (TOST) for dependent samples [36]. Accordingly, a between-mean effect size is regarded as equivalent to 0 if falling within the upper and lower equivalence bounds. In such a case, both equivalence bounds will yield a *p* < 0.05. Sample size estimation for this procedure, performed via the R package *TOSTER* (https://cran.r-project.org/web/packages/TOSTER/TOSTER.pdf) [37], yielded a minimum of *N* = 52 observations for detecting equivalence with a 95% power, α = 0.05 and upper and lower equivalence bounds of  – 0.5 and 0.5, respectively.

Convergence between the t-FAB-M/-V and its in-person version was tested in both HPs and clinical groups via correlational techniques. Sample size estimation for such analyses, as performed via the R package *pwr* (https://CRAN.R-project.org/package=pwr), yielded a minimum *N* of 20 by addressing a one-tailed hypothesis testing (i.e., positive correlation) at α = 0.05, an expected medium-to-large effect size ρ = 0.5 and an 80% power.

Norms were derived through the Equivalent Score (ES) method [38, 39]; outer and inner tolerance limits (TLs), as well as ES thresholds, were computed on ranked scores adjusted for significant anagraphic–demographic confounders via a stepwise regression procedure. The quasi-continuous ES scale allows for clinical judgments as follows: ES = 0 (adjusted scores lower than the outer TL) indexing an “impaired” performance; ES = 1, which indicates a “borderline” score; ES = 2–3-4 indicate a “normal” score. To derive norms, consistently with previous studies [2] and by adopting the R package *pwr* (https://CRAN.R-project.org/package=pwr), a minimum sample size of 287 participants was deemed as adequate, by addressing a small-to-medium effect size (*f*^2^ = 0.05), with a 95% power and α = 0.05, within a multiple regression model (*df*_numerator_ = 3).

Diagnostic accuracy was tested via receiver-operating characteristics (ROC) analyses by addressing t-FAB scores of the whole clinical group (stroke, EPD, SVD, MD) against the whole normative sample. For this single-test ROC analysis, the minimum sample sizes for the normative and clinical groups were estimated, according to Obuchowski [40], by means of *easyROC* (http://www.biosoft.hacettepe.edu.tr/easyROC/), at *N* = 190 and *N* = 19, respectively, a case–control allocation ratio of 10 and to detect an AUC = 0.7 with a power of 90% and α = 0.05.

Diagnostic accuracy was further explored for descriptive purposes (due to the small sample sizes addressed) by comparing (1) each clinical subgroups against the normative sample and (2), within the whole clinical cohort, t-FAB scores of participants performing defectively (i.e., ES = 0/1) on the in-person FAB against those performing within the normal range (i.e., ES ≥ 2). As to ROC analyses addressing the SVD group, due to the high heterogeneity in cognitive status as assessed by the MMSE, participants were subdivided into those obtaining an MMSE ES ≤ 1 (impaired/borderline performance; *N* = 6) and those obtaining an MMSE ES > 1 (normal performance; *N* = 14) [41].

## Results

### Standardization

Normative sample stratification is shown in Table 1. HPs’ background and cognitive profiles are summarized in Table 2. Ceiling effects at the t-FAB (i.e., a score of 12/12) were detected in 35.8% (t-FAB-M) and 39.3% (t-FAB-V) of HPs. Acceptability rate was of 100%.

**Table 1:** Sample stratification for age, education, and sex

Male/female	Age						
Education	30 ≤	31–45	46–60	61–70	71–80	≥ 81	Total
5 ≤	0/0	0/0	0/0	1/2	1/2	1/8	3/12
6–8	5/0	2/2	8/13	4/2	3/4	2/3	24/24
9–12	1/1	0/1	2/12	4/5	3/2	1/1	11/22
13–16	33/29	9/11	21/43	5/13	1/3	0/1	69/100
≥ 17	13/20	4/5	9/11	7/7	2/0	1/2	36/45
Total	52/50	15/19	40/79	21/29	10/11	5/15	143/203

**Table 2:** Demographic and cognitive data of the normative sample

*N*	346
Age (years)	48.57 ± 18.92 (18–96)
Sex (M/F)	143/203
Education (years)	13.42 ± 3.76 (4–23)
*N* for Italian regions	
North Italy	81.8%
Center Italy	4.9%
South Italy	13.3%
*N* for occupation	
Predominantly manual	24.2%
Manual/clerical	24.2%
Predominantly clerical	51.6%
Itel-MMSE (*N* = 253)	21.58 ± .82 (17–22)
PVF (*N* = 330)	42.58 ± 13.45 (13–85)
SVF (*N* = 330)	55.54 ± 13.17 (3–84)
AVF (*N* = 330)	42.19 ± 13.67 (4–74)
CSI (*N* = 330)	.86 ± .22 (.1–2.22)
BDS-WM (*N* = 277)	4.76 ± 1.25 (0–6)
BDS-T (*N* = 277)	4.94 ± 2.01 (0–8)
t-FAB	
t-FAB-M	10.84 ± 1.46 (2–12)
t-FAB-V	10.98 ± 1.24 (4–12)
t-FAB-1	5.22 ± .86 (2–6)
t-FAB-2-M	5.62 ± .97 (0–6)
t-FAB-2-V	5.77 ± .73 (0–6)

*N*  number of participants, *M*  male, *F*  female, *Itel-MMSE*  Italian telephone-based Mini-Mental State Examination, *PVF*  phonemic verbal fluency, *SVF*  semantic verbal fluency, *AVF*  alternate verbal fluency, *CSI*  Cognitive Shifting Index, *BDS-WM*  backward digit span—working memory, *BDS-T*  backward digit span—total, *t-FAB*  telephone-based Frontal Assessment Battery, *t-FAB-M*  motor-mediated t-FAB, *t-FAB-V*  verbal-mediated t-FAB, *t-FAB-1*  tasks *Conceptualization* + *Mental flexibility*, *t-FAB-2-M/V*  tasks *Sensitivity to interference* + *Inhibitory control*

Construct validity data with the other telephone-based measures are displayed in Table 3. Each t-FAB score converged with the vast majority of the other telephone-based test scores, the highest associations being found with PVF, SVF and AVF scores. t-FAB total and subtests scores were all internally related at α_adjusted_ = 0.017 (t-FAB-M: 0.19 ≤ *r*_*s*_(346) ≤ 0.9, *p* < 0.001; t-FAB-V: 0.15 ≤ *r*_*s*_(346) ≤ 0.84, *p* ≤ 0.005).

**Table 3:** Spearman’s correlation coefficients between t-FAB scores and construct validity measures

	t-FAB-M	t-FAB-V	t-FAB-1	t-FAB-2-M	t-FAB-2-V
BDS-T	*r*_*s*_(277) = .22*	*r*_*s*_(277) = .21*	*r*_*s*_(277) = .2*	*r*_*s*_(277) = .17†	*r*_*s*_(277) = .14†
BDS-WM	*r*_*s*_(277) = .21*	*r*_*s*_(277) = .21*	*r*_*s*_(277) = .19†	*r*_*s*_(277) = .16†	*r*_*s*_(277) = .16†
PVF	*r*_*s*_(330) = .3*	*r*_*s*_(330) = .28*	*r*_*s*_(330) = .28*	*r*_*s*_(330) = .23*	*r*_*s*_(330) = .14†
SVF	*r*_*s*_(330) = .32*	*r*_*s*_(330) = .25*	*r*_*s*_(330) = .24*	*r*_*s*_(330) = .3*	*r*_*s*_(330) = .15†
AVF	*r*_*s*_(330) = .39*	*r*_*s*_(330) = .32*	*r*_*s*_(330) = .29*	*r*_*s*_(330) = .33*	*r*_*s*_(330) = .22*
CSI	*r*_*s*_(330) = .2*	*r*_*s*_(330) = .18*	*r*_*s*_(330) = .14†	*r*_*s*_(330) = .19*	*r*_*s*_(330) = .19*
Itel-MMSE	*r*_*s*_(253) = .22*	*r*_*s*_(253) = .19†	*r*_*s*_(253) = .14†	*r*_*s*_(253) = .24*	*r*_*s*_(253) = .23*

†Significant at α = .05; *significant at α_adjusted_ = .0013, *t-FAB*  telephone-based Frontal Assessment Battery, *t-FAB-M*  motor-mediated t-FAB, *t-FAB-V*  verbal-mediated t-FAB, *FAB-1*  tasks *Conceptualization* + *Mental flexibility*, *t-FAB-2-M/V*  tasks *Sensitivity to interference* + *Inhibitory control*, *BDS-WM*  backward digit span—working memory, *BDS-T*  backward digit span—total, *PVF*  phonemic verbal fluency, *SVF*  semantic verbal fluency, *AVF*  alternate verbal fluency, *CSI*  cognitive shifting index, *Itel-MMSE*  Italian telephone-based Mini-Mental State Examination

Inter-rater and test–retest reliability were high for both t-FAB-M (test–retest: ICC = 0.88; and ICC = 0.84) and t-FAB-V (test–retest: ICC = 0.94; inter-rater: ICC = 0.78). A clear mono-component factor (reflecting “frontal-executive global efficiency”) underpinned both the t-FAB-M (39.5% of variance explained; loading *range* = 0.54-0.67) and t-FAB-V (34.2% of variance explained; loading *range* = 0.43-0.66).

Despite t-FAB-M scores (*M* = 10.8; *SD* = 1.46) being lower than t-FAB-V (11 ± 1.23; *t*_345_ = -− 3.3; *p* = 0.001), the two versions showed statistical equivalence at the TOST procedure (both upper and lower equivalence bound yielding a *p* < 0.001).

The in-person FAB converged, at α_adjusted_ = 0.0013, with both the t-FAB-M (*r*_*s*_(20) = 0.78; *p* < 0.001) and t-FAB-V (*r*_*s*_(20) = 0.75; *p* < 0.001) when administered at the 14-day follow-up, as well as at the 48-h follow-up (t-FAB-M: *r*_*s*_(50) = 0.44; *p* = 0.001; t-FAB-V: *r*_*s*_(50) = 0.46; *p* = 0.001).

Education predicted all t-FAB scores (*p* < 0.001), whereas age only t-FAB-M and t-FAB-2-M scores (*p* ≤ 0.004). Selected correction factors, TLs and ES thresholds are shown in Table 4. An automated correction sheet is provided in the Supplementary Material 2.

**Table 4:** Adjustment grids and Equivalent Scores (ES) for t-FAB scores

	Education						
		5	8	11	13	16	18
	**Age**						
t-FAB-M	35	1.17	0.46	– 0.02	– 0.27	– 0.59	– 0.77
	40	1.21	0.50	0.02	– 0.23	– 0.55	– 0.72
	45	1.27	0.56	0.08	– 0.18	– 0.49	– 0.67
	50	1.34	0.63	0.14	– 0.11	– 0.42	– 0.60
	55	1.42	0.71	0.23	– 0.03	– 0.34	– 0.52
	60	1.52	0.81	0.33	0.07	– 0.24	– 0.42
	65	1.64	0.92	0.44	0.19	– 0.12	– 0.30
	70	1.77	1.06	0.58	0.33	0.01	– 0.17
	75	1.93	1.22	0.74	0.48	0.17	– 0.01
	80	2.11	1.40	0.92	0.66	0.35	0.17
	85	2.32	1.60	1.12	0.87	0.55	0.38
	90	2.54	1.83	1.35	1.10	0.78	0.61
t-FAB-V		1.83	0.65	0.12	– 0.10	– 0.32	– 0.43
t-FAB-1		0.96	0.48	0.15	– 0.02	– 0.23	– 0.35
t-FAB-2-M	35	0.47	0.17	– 0.03	– 0.13	– 0.27	– 0.34
	40	0.49	0.19	– 0.01	– 0.11	– 0.25	– 0.32
	45	0.52	0.22	0.02	– 0.09	– 0.22	– 0.29
	50	0.55	0.26	0.05	– 0.05	– 0.18	– 0.26
	55	0.60	0.30	0.09	– 0.01	– 0.14	– 0.22
	60	0.65	0.35	0.14	0.04	– 0.09	– 0.17
	65	0.70	0.41	0.20	0.10	– 0.03	– 0.11
	70	0.77	0.47	0.27	0.17	0.03	– 0.04
	75	0.85	0.55	0.35	0.24	0.11	0.04
	80	0.94	0.64	0.44	0.33	0.20	0.13
	85	1.04	0.74	0.54	0.44	0.30	0.23
	90	1.16	0.86	0.66	0.55	0.42	0.34
t-FAB-2-V		0.69	0.25	0.05	– 0.04	– 0.12	– 0.16

	Equivalent Scores						
	oTL	iTL	0	1	2	3	4
t-FAB-M	8.07	8.77	≤ 8.07	8.08–9.45	9.46–10.5	10.51–11.03	≥ 11.04
t-FAB-V	8.48	9	≤ 8.48	8.49–9.83	9.84–10.68	10.69–10.93	≥ 10.94
t-FAB-1	3.54	4.07	≤ 3.54	3.55–4.49	4.50–4.84	4.85–5.45	≥ 5.46
t-FAB-2-M	3.16	4	≤ 3.16	3.17–4.90	4.91–5.66	5.67–5.84	≥ 5.85
t-FAB-2-V	3	5	≤ 3	3.01–5.77	5.78–5.86	5.87–5.99	≥ 6

t-FAB = telephone-based Frontal Assessment Battery, t-FAB-M = motor-mediated t-FAB, t-FAB-V = verbal-mediated t-FAB, FAB-1 = tasks *Conceptualization* + *Mental flexibility*, t-FAB-2-M/V = tasks *Sensitivity to interference* + *Inhibitory control*, *AS* adjusted score, *iTL* inner tolerance limit, *oTL* outer tolerance limit, *RS* raw score

t-FAB-M: AS = RS + 0.000002*[(age^3)−167,162.855491]−3.484874 *[log10(education)−1.106787]

t-FAB-V: AS = RS + 15.62586*(1/education−0.083175)

t-FAB-1: AS = RS −1.020062*[ln(education)−2.548471]

t-FAB-2-M: AS = RS + 0.000001*[(age^3)−167,162.855491]−1.462853*[log10(education)−1.106787)

t-FAB-2-V: AS = RS + 5.921931*(1/education−0.083175)

Adjustment factors have been extracted from the above adjustment equations and do not always reflect empirical co-occurrences. Supplementary Material 1 provides a sheet for the automated computation of ASs

### Clinical usability

Out of 67 individuals with neurological conditions consecutively screened for eligibility at the neuropsychology clinics, 10 refused to participate without further explanations, 4 stated not to be available due to working activities, 7 were excluded as being deemed by their respective caregivers as not to be able to participate due to severe behavioral alterations that would have undermined compliance, 3 were unable to execute tasks due to severe hearing deficits and 3 presented with severe medical-general conditions. Background, clinical and cognitive data of the final clinical cohort (*N* = 40) are reported in Table 5. Within the whole clinical group, FAB scores correlated (α_adjusted_ = 0.025) with both t-FAB-M (*r*_*s*_(33) = 0.62; *p* < 0.001) and t-FAB-V (*r*_*s*_(33) = 0.43; *p* = 0.012) scores. When discriminating the whole clinical group from the normative sample, moderate-to-high accuracy was detected for both the t-FAB-M (AUC = 0.76; *SE* = 0.05; 95% CI [0.66, 0.85]) and t-FAB-V (AUC = 0.73; *SE* = 0.05; 95% CI [0.63, 0.83]).

**Table 5:** Demographic, clinical, and cognitive data of participants with neurological diseases

	Total	EPD	SVD		Stroke		MD
			MMSE ES > 1	MMSE ES ≤ 1	Left	Right	
*N*	40	6	14	6	5	5	4
Sex (M/F)	17/23	4/2	5/9	2/4	3/2	2/3	1/3
Age (years)	73.1 ± 11.5 (38–91)	76 ± 5.06 (70–82)	76.07 ± 8.87 (56–87)	81.33 ± 7.97 (68–91)	54.6 ± 12.42 (38–67)	68.8 ± 9.73 (54–78)	74.5 ± 9.98 (60–82)
Education (years)	13.3 ± 4.39 (5–18)	14 ± 5.18 (5–18)	12 ± 4.37 (5–18)	14.5 ± 3.94 (8–18)	14 ± 4.18 (8–18)	13.8 ± 5.31 (8–18)	13.5 ± 4.8 (8–18)
MMSE (*N* = 38)							
Raw scores	27.26 ± 2.84 (20–30)	29 ± 1.27 (27–30)	28.71 ± 1.27 (26–30)	23.33 ± 1.75 (21–26)	30 ± 0 (30)	26 ± 1.87 (23–28)	25 ± 4.08 (20–30)
ES ≤ 1		1	–	–	–	2	3
FAB (*N* = 33)							
Raw scores	14.45 ± 3.09 (5–18)	15 ± 1.68 (13–17)	15.77 ± 1.79 (13–18)	12.8 ± 5.17 (5–17)	13 ± 0 (13)	12.25 ± 3.3 (8–16)	14 ± 4.2 (9–18)
ES ≤ 1		–	–	2	1	3	2
t-FAB-M	8.63 ± 2.66 (4–12)	9 ± 3.23 (4–12)	9.43 ± 1.99 (6–12)	7.5 ± 3.89 (4–12)	10 ± 2.35 (6–12)	7.4 ± 1.67 (6–10)	6.75 ± 2.22 (4–9)
t-FAB-V	9.3 ± 2.17 (4–12)	9.5 ± 3.02 (4–12)	10 ± 1.71 (7–12)	8.67 ± 2.25 (7–12)	9.8 ± 1.79 (8–12)	9.4 ± 1.67 (8–12)	6.75 ± 2.22 (4–9)

*N*  number of participants, *M*  male, *F*  female, *EPD*  extra-pyramidal disease, *SVD*  small vessel disease, *MD*  mixed dementia, *ES*  Equivalent Score, *MMSE*  Mini-Mental State Examination, *FAB*  Frontal Assessment Battery, *t*-*FAB*  telephone-based Frontal Assessment Battery, *t-FAB-M*  motor-mediated t-FAB, *t-FAB-V*  verbal-mediated t-FAB

As to descriptive ROC analyses, AUC values were overall moderate-to-high (AUC = 0.65-0.97) for each clinical group and similar between the t-FAB-M and t-FAB-V (Table 6). Moreover, when descriptively comparing, within the whole clinical group, patients with an ES = 0/1 on the FAB vs*.* those with an ES ≥ 2, moderate-to-high accuracy yielded with respect to both t-FAB-M (AUC = 0.83; *SE* = 0.09; 95% CI [0.66, 0.1]) and t-FAB-V (AUC = 0.7; *SE* = 0.11; 95% CI [0.49, 0.91]).

**Table 6:** ROC analysis for each clinical group against the normative sample

		AUC	*SE*	CI 95%
Stroke (*N* = 10)	t-FAB-M	.78	.08	[.62, .95]
	t-FAB-V	.74	.01	[.56, .93]
Left (*N* = 5)	t-FAB-M	.62	.13	[.37, .88]
	t-FAB-V	.71	.14	[.45, .97]
Right (*N* = 5)	t-FAB-M	.94	.03	[.88, 1]
	t-FAB-V	.78	.13	[.51, 1]
EPD (*N* = 6)	t-FAB-M	.68	.12	[.44, 92]
	t-FAB-V	.65	.12	[.42,.88]
SVD (*N* = 20)	t-FAB-M	.73	.07	[.59, .86]
	t-FAB-V	.7	.07	[.56, .85]
MMSE ES > 1 (*N* = 14)	t-FAB-M	.71	.09	[.55, .88]
	t-FAB-V	.67	.09	[.5, .84]
MMSE ES ≤ 1 (*N* = 6)	t-FAB-M	.76	.13	[.51, .1]
	t-FAB-V	.78	.13	[.54, .1]
MD (*N* = 4)	t-FAB-M	.96	.02	[.92, 1]
	t-FAB-V	.97	.02	[.93, 1]

*ROC*  receiver-operating characteristics, *N*  number of participants, *AUC*  area under the curve, *SE*  standard error, *EPD*  extra-pyramidal disorder, *SVD*  small vessel disease, *MD*  mixed dementia, *ES*  Equivalent Score, *MMSE*  Mini-Mental State Examination, *FAB*  Frontal Assessment Battery, *t-FAB*  telephone-based Frontal Assessment Battery, *M*  motor-mediated t-FAB, *V*  verbal-mediated t-FAB

## Discussion

The present study provides Italian clinicians and researchers with a statistically sound standardization of a TBCS test for frontal-executive deficits, namely the t-FAB, along with evidence of its clinical usability in neurological populations. This study is unprecedented not only within the Italian literature but also within the international ones, and it enriches the range of standardized TBCS instruments already available in Italy [2, 15].

As far as to its standardization in HPs, the t-FAB comes with regression-based norms, as well as with evidence on convergent validity, internal validity among its scores, a solid underlying factorial structure, high inter-rater and test–retest reliability and invariance as compared to its in-person version. With respect to its clinical usability, the present preliminary evidence suggests that the t-FAB is able to accurately discriminate neurological cases from healthy controls, identifying frontal-executive disturbances in neurological individuals as the in-person FAB does.

The t-FAB comes with two administration versions as to *Sensitivity to interference* and *Inhibitory control* tasks, one requiring motor and the other verbal responses (t-FAB-M and t-FAB-V, respectively). Although HPs reported lower scores on the t-FAB-M version as compared to the t-FAB-V, both versions showed statistical equivalence. Moreover, norms have been provided separately for each version, this allowing a flexible administration of the two versions according to the participant’s clinical features. For instance, the t-FAB-M is more appropriate for individuals with motor speech disorders (e.g., dysarthria/anarthria) but spared hand movements, and vice versa with respect to the t-FAB-V. For the same reasons, the two t-FAB versions could be administered as parallel versions when performing follow-ups.

The slight discrepancies in norms between the t-FAB-M and t-FAB-V are likely due to the fact that inhibition of motor responses is more demanding than that of verbal responses [42]. This proposal is supported by the finding that age negatively predicts the t-FAB-M scores only, in accordance with the notion of motor inhibition being more affected than verbal inhibition with advancing age [43]. Notably, the influence of response modalities on inhibitory-related tasks has been proposed to be negligible in clinical populations, where inhibitory process impairment is likely widespread [44]. This further supports the adoption of the t-FAB as a valid screener of frontal-executive functioning in different neurological diseases. In fact, findings herewith reported as to the diagnostic accuracy of the two t-FAB versions show that the t-FAB-M and t-FAB-V are both able to identify frontal-executive dysfunctions in different neurological populations.

There are some limits of the present study that need to be acknowledged. First, the clinical usability of the t-FAB needs to be further verified in larger neurological samples, in particular focusing on diseases with predominant frontal involvement (e.g., motor neuron disease–frontotemporal degeneration *spectrum* disorders, acquired focal damages of frontal areas and networks due to traumatic, neoplastic, demyelinating, metabolic or infectious etiologies). Such an issue has to be particularly underlined as to those ROC analyses performed on clinical subsamples (Table 6), which should be treated as purely descriptive and preliminary. Second, further in-depth investigations are needed to assess the diagnostic properties, sensitivity to disease severity, responsiveness and reliable change of the t-FAB in neurological and geriatric cohorts [13]. Finally, with respect to the present stroke cohort, the discrepancies found in diagnostic accuracy between the t-FAB-M and t-FAB-V based on lesion side cannot be properly explored due to the small sample size of individuals presenting with either right- or left-lateralized damaged (*N* = 5 each).

In conclusion, the present work offers the first telephone-based version of FAB, a normed, valid, reliable and clinically usable TBCS test for screening frontal-executive functioning, encouraging its adoption in both clinical practice and research settings in neurology and geriatrics.

## Supplementary Information

Below is the link to the electronic supplementary material.

Supplementary file1 (DOCX 18 KB)

Supplementary file2 (XLSX 80 KB)

## Data Availability

Data collected and analyzed within the present study are openly accessible at https://osf.io/6v4bu/.
