# Supplementary material for: Telephone-based Frontal Assessment Battery (t-FAB): standardization for the Italian population and clinical usability in neurological diseases
Source: Aging Clin Exp Res. 2022 Jun 14;34(7):1635–44. doi: 10.1007/s40520-022-02155-3 (PMC9194888; doi:10.1007/s40520-022-02155-3)
Supplement: Supplementary file 1 — Supplementary file1 (DOCX 18 KB) [file 40520_2022_2155_MOESM1_ESM.docx]

**Telephone-based Frontal Assessment Battery (t-FAB)^[[1]](#footnote-1)^**

| **N.B.:** gli *item* “a” si riferiscono alla versione M (“motor responses”), mentre gli *item* “b” si riferiscono alla versione V (“verbal responses”. È possibile somministrare sia la versione t-FAB-M che t-FAB-V o entrambe (in quest’ultimo caso, nell’ordine in cui appaiono). | | | | |
| --- | --- | --- | --- | --- |
| **1.** **1. “**In che cosa sono simili una banana e un’arancia?”. Risposta: ________ (“frutta”=1 pt.)  ***Note:*** in caso di fallimento totale *“*non sono simili*”* o di fallimento parziale *“*entrambe hanno la  buccia*”* aiutare il paziente: *“*la banana e l’arancia sono entrambe [*…*]*”* ma assegnare 0 a  questa risposta; non aiutare per i successivi due *item*.  **1.2.** In che cosa sono simili un tavolo e una sedia? Risposta: ________ (“mobili”=1 pt.)  **1.3.** In che cosa sono simili un tulipano, una rosa e una margherita? Risposta: ________ (“fiori”=1 pt.) | | | | [__/3] |
| **2.** = *numero di parole prodotte alle fluenze fonemiche “S”*: ___  **Attribuzione del punteggio**: 3=>9 parole; 2=6-9 parole; 1=3-5 parole; 0=3< parole | | | | [__/3] |
| **3.a.***“*Batta due volte sul tavolo quando io batto una volta.*”*. ***Note*:** per essere sicuri che il soggetto abbia capito le istruzioni, si effettua una serie di 3 prove: “1-1-1”; Risposta: __-__-__  “Batta una volta sul tavolo quando io batto due volte.*”.* ***Note*:** per essere sicuri che il soggetto abbia capito le istruzioni, si effettua una serie di 3 prove: “2-2-2”; Risposta: __-__-__  Effettuare la seguente serie: 1-1-2-1-2-2-2-1-1-2; Risposta: __-__-__-__-__-__-__-__-__-__  **Attribuzione del punteggio**: 3=0 errori; 2=1-2 errori; 1=3 errori; 0=>3 errori. | | | | [__/3] |
| **4.a.***“*Batta una volta sul tavolo quando io batto una volta.*”*. ***Note*:** per essere sicuri che il soggetto abbia capito le istruzioni, si effettua una serie di 3 prove: “1-1-1”; Risposta: __-__-__  “Non batta sul tavolo quando io batto due volte.*”.* ***Note*:** per essere sicuri che il soggetto abbia capito le istruzioni, si effettua una serie di 3 prove: “2-2-2”; Risposta: __-__-__  Effettuare la seguente serie: 1-1-2-1-2-2-2-1-1-2; Risposta: __-__-__-__-__-__-__-__-__-__  **Attribuzione del punteggio**: 3=0 errori; 2=1-2 errori; 1=3 errori; 0=>3 errori. | | | | [__/3] |
| **3.b.***“*Dica «due» quando io dico «uno»”. ***Note*:** per essere sicuri che il soggetto abbia capito le istruzioni, si effettua una serie di 3 prove: “1-1-1”; Risposta: __-__-__  “Dica «uno» quando io dico «due»*”.* ***Note*:** per essere sicuri che il soggetto abbia capito le istruzioni, si effettua una serie di 3 prove: “2-2-2”; Risposta: __-__-__  Effettuare la seguente serie: 1-1-2-1-2-2-2-1-1-2; Risposta: __-__-__-__-__-__-__-__-__-__  **Attribuzione del punteggio**: 3=0 errori; 2=1-2 errori; 1=3 errori; 0=>3 errori. | | | | [__/3] |
| **4.b.***“*Dica «uno» quando io dico «uno»”. ***Note*:** per essere sicuri che il soggetto abbia capito le istruzioni, si effettua una serie di 3 prove: “1-1-1”; Risposta: __-__-__  “Non dica niente quando io dico «due»*”.* ***Note*:** per essere sicuri che il soggetto abbia capito le istruzioni, si effettua una serie di 3 prove: “2-2-2”; Risposta: __-__-__  Effettuare la seguente serie: 1-1-2-1-2-2-2-1-1-2; Risposta: __-__-__-__-__-__-__-__-__-__  **Attribuzione del punteggio**: 3=0 errori; 2=1-2 errori; 1=3 errori; 0=>3 errori. | | | | [__/3] |
|  | **PG** | **PC** | **PE** | |
| **t-FAB-1** | ___/6 | ___ | ___ | |
| **t-FAB-M-2** | ___/6 | ___ | ___ | |
| **t-FAB-V-2** | ___/6 | ___ | ___ | |
| **t-FAB-M** | ___/12 | ___ | ___ | |
| **t-FAB-V** | ___/12 | ___ | ___ | |

1. Aiello, E. N., Esposito, A., Gramegna, C., Gazzaniga, V., Difonzo, T., Zago, S., Appollonio, I. M., & Bolognini, N. (2022). The Frontal Assessment Battery (FAB) and its sub-scales: validation and updated normative data in an Italian population sample. *Neurological Sciences, 43*, 979–984. <https://doi.org/10.1007/s10072-021-05392-y> [↑](#footnote-ref-1)
